# A prospective, multicenter, open-label study of the clinical efficacy of tapentadol extended-release in the treatment of cancer-related pain and improvement in the quality of life of opioid-naïve or opioid-resistant patients

**DOI:** 10.1007/s00520-022-06992-w

**Published:** 2022-04-14

**Authors:** Ji Yoon Jung, Hong Jae Chon, Young Jin Choi, Sang Eun Yeon, Seok Young Choi, Kyung Hee Lee

**Affiliations:** 1 Department of Hematology-Oncology, College of Medicine, Yeungnam University, Daegu, Korea; 2 Medical Oncology, CHA Bundang Medical Center, CHA University School of Medicine, Seongnam, Gyeonggi-do Korea; 3 Department of Internal Medicine, Pusan National University School of Medicine, Busan, Korea; 4 Medical Affairs, Janssen Korea Ltd, Seoul, Korea

**Keywords:** Tapentadol, Chronic pain, Cancer pain, Real-world data, Korean

## Abstract

**Purpose:**

This study aimed to investigate the clinical efficacy of tapentadol extended-release (ER) on pain control and the quality of life (QoL) of patients with moderate to severe chronic cancer pain in clinical practice in Korea.

**Methods:**

In this prospective, open-label, multicenter trial, patients with sustained cancer pain as well as chronic pain, who were or were not using other analgesics were enrolled. Thirteen centers recorded a total of 752 patients during the 6-month observation period, based on the tapentadol ER dose and tolerability, prior and concomitant analgesic treatment, pain intensity, type of pain, adverse effects, and clinical global impression change (CGI-C). Of those 752 patients, 688 were enrolled, and 650 completed the study for efficacy and adverse drug reactions; among them, 349 were cancer patients.

**Results:**

Tapentadol ER significantly reduced the mean pain intensity including neuropathic pain during the observation period by 2.9 points (from a mean 7 ± 0.87 to 4.1 ± 2.02). Furthermore, QoL was observed to be significantly improved based on the CGI-C, an objective measure.

**Conclusion:**

This study showed that tapentadol ER was effective for treating patients with moderate to severe cancer pain and neuropathic pain, and therefore it significantly improved the patients’ QoL.

## Introduction

Pain is the most common symptom in cancer patients. Up to two-thirds of patients experience pain, and with tumor progression, the pain worsens. More than 40% of cancer patients have moderate to severe pain [1–3], an important factor that influences the quality of life (QoL) of these patients. However, most cancer patients do not receive adequate treatment for pain owing to the difficulty in pain management [4, 5]. This is mainly because cancer pain is a mixed type of pain arising from nociceptive and neuropathic pain [6, 7]. In a previous study on cancer patients, 60% complained of nociceptive pain, 20% of neuropathic pain, and the remaining 20% complained of a combination of neuropathic and nociceptive pain [8]. It has been reported that although cancer itself causes neuropathic pain, treatments such as surgery, chemotherapy, and radiation therapy also contribute to neuropathic pain. In particular, some patients do not receive adequate chemotherapy owing to chemotherapy-induced peripheral neuropathy [9–11]. Therefore, pain control is as important as frontline treatment for cancer patients, in particular the control of neuropathic pain [12, 13]. The World Health Organization (WHO) recommends aggressive control of cancer pain and suggests a three-step type of pharmacological management called the "analgesic ladder," which includes the administration of weak to strong opioids [13]. However, according to a recent report, many experts recommend using strong opioid analgesics as soon as possible for moderate to severe pain, together with anti-neuropathic pain drugs, such as pregabalin [14]. Despite these recommendations, it is difficult to control cancer pain along with neuropathic pain as many studies have failed to show promising results. In addition, a decrease in compliance to a particular drug when other classes of drugs are added is also an important factor for treatment failure [15–19].

Tapentadol extended-release (ER) is a dual-acting mu-opioid receptor agonist and noradrenaline reuptake inhibitor. These two mechanisms of action contribute to the regulation of nociceptive and neuropathic pain. Due to the synergistic effect of these separate mechanisms, this drug has increased ability to control pain, decreased side effects, and shows good patient compliance [20, 21]. Several clinical trials have demonstrated the efficacy and safety of tapentadol ER in nonmalignant patients when compared with that of other types of opioid analgesics, such as morphine or oxycodone, and there have also been reports of its use in treating cancer pain [22–27]. However, the majority of these studies were conducted with < 200 patients, and there are only a few large-scale studies on the improvement of the QoL of patients following the improvement of neuropathic pain. Therefore, further research is required to accurately evaluate the efficacy of these drugs and their improvement of QoL.

We conducted a prospective, multicenter, open-label, large-scale study with a total of 650 participants, including 349 cancer patients, and we aimed to analyze the improvement of patients' QoL according to the improvement of nociceptive pain and neuropathic pain. We evaluated this improvement in opioid-naïve or opioid-resistant patients using an objective index: the clinical global impression change (CGI-C) [28–30]. The primary endpoint was pain intensity difference (PID), and the secondary endpoint was the CGI-C and safety.

## Patients and methods

This prospective, multicenter, open-label, observational study was conducted at 13 centers in South Korea from October 2017 to May 2020. This study was conducted according to the Declaration of Helsinki, and the study protocol was approved by the respective Institutional Review Board of each center.

### Patients

This study enrolled patients ≥ 18 years of age for whom tapentadol ER had been newly prescribed for the treatment of severe chronic pain, including the cancer-related pain (Numeric Rating Scale (NRS) score ≥ 7 or NRS score ≠ 0, even with the use of WHO ladder II or high drugs) based on the locally approved label. Patients with contraindications for tapentadol, such as a history of hypersensitivity to tapentadol or any component of the product; with significant respiratory depression, acute or severe bronchial asthma, or hypercapnia with mu-opioid receptor agonist; with paralytic ileus; with acute intoxication with alcohol, hypnotics, centrally acting analgesics, or psychotropic drugs; who received monoamine oxidase inhibitors or had received them within the last 14 days; and with genetic problems, such as galactose intolerance, Lapp lactase deficiency, or glucose-galactose malabsorption were excluded from the study. Patients who signed informed consent to the use of personal information in connection with, and were willing to participate in, post-marketing surveillance were included for the analysis.

### Study design

The primary objective of this study was to evaluate the effect of tapentadol ER in opioid-naïve or opioid-resistant patients in Korea with chronic pain, including cancer pain, which was not sufficiently controlled in clinical practice. The effect of the drug on neuropathic pain as well as nociceptive pain was investigated in detail. The second objective of this study was to evaluate the improvement in patients' QoL due to tapentadol ER; this evaluation was conducted using the CGI-C, which was assessed by the physicians who participated in the study.

After baseline evaluation (visit 1), tapentadol ER was administered to patients. Next, the efficacy, CGI-C, and adverse drug reactions were assessed at 4 (visit 2), 12 (visit 3), and 24 weeks (visit 4), respectively. During the baseline study, previous drug usage was investigated, particularly that of opioid analgesics. In opioid-resistant patients who required conversion to tapentadol ER, all other opioids were discontinued before tapentadol ER was initiated, considering the equivalent dose according to the National Comprehensive Cancer Network guidelines. In opioid-naïve patients, drug administration was initiated with a dose of 50 mg twice daily. After treatment initiation, the dose was individually titrated under the close supervision of the prescribing physician at a level that produced an adequate analgesic effect and minimized the side effects. According to the clinical trial data, the method of increasing the dose by 50 mg every 3 days while maintaining the administration twice daily was observed to be an appropriate titration method. This drug has not been studied in daily doses exceeding 500 mg; hence, this drug is not recommended for such usage [31, 32].

All adverse drug reactions and special situations following exposure to a product in the study were systematically recorded, regardless of the seriousness or causality. Adverse drug reactions were recorded at the first use of tapentadol ER within the study period and applied to all adverse events that occurred within 30 days after a patient’s last use of tapentadol ER.

### Assessment

The effective outcome measure for this study was average pain intensity over the preceding 24 h. At each visit, the patients were asked to indicate their pain score using the 11-point NRS, where a score of 0 indicated “no pain” and a score of 10 indicated “pain as bad as you can imagine.” Effectiveness was assessed by the percentage of patients achieving various levels of improvement in pain intensity from baseline at various time points. Disease severity was evaluated based on the NRS score. Each visit and the change from baseline to the final assessment of pain intensity (11-point NRS) was summarized using the number, mean, standard deviation (SD), median, minimum (min), and maximum (max) values. For analyzing the proportion of response, the change from baseline to each visit or the final assessment was calculated. The %PID30 and %PID50 (defined as a 30% and 50% reduction in pain intensity from baseline) were also calculated using 95% two-sided confidence intervals (CI).

$$\% {\text{ Pain Intensity Difference }} = \frac{{({\text{NRS score }}\left( {\text{Visit 1}} \right) - {\text{NRS score }}\left( {{\text{Visit 2}},{ 3},{\text{ 4 or final assessment}}} \right)}}{{{\text{NRS score }}\left( {\text{Visit 1}} \right)}} \times {1}00$$

Neuropathic pain was evaluated as “yes,” “none,” or “unknown” by the investigator directly interviewing the patient at each visit. Questions about neuropathic pain were asked by the investigator to the patient personally, and there were no special regulations or questions.

The CGI-C was assessed by the clinician who responded to the question “compared with the patient’s condition at baseline, how has it changed?” using 1 of 7 possible responses: very much improved = 1, much improved = 2, minimally improved = 3, no change = 4, minimally worse = 5, much worse = 6, very much worse = 7. Effectiveness was assessed by the percentage of patients achieving “no change = 4” or higher. The CGI-C assessments were summarized descriptively by presenting the number and percentage of participants in each category. In addition, the percentage of participants with “no change” or higher improvement was summarized. All findings from the examinations were medically adjudicated, interpreted, and described. The missing data correction for the effectiveness evaluation at the baseline was not executed. When data was missing from the NRS or CGI-C data on other visits, the last observation carried forward (LOCF) method was used.

### Statistical analyses

The Wilcoxon signed-rank test was applied to test the statistical significance of the difference of NRS scores between the baseline (visit 1) and subsequent visits (visit 2, visit 3, and visit 4). To test the statistical significance of the CGI-C at each subsequent visit, which is the difference value itself, the range of CGI-C was modified to {-3, 3} from {1, 7} by modifying “very much worse” to be − 3 instead of 7, “no change” to be 0 instead of 4, and “very much improved” to be 3 instead of 1. This modification was done by calculating (4 − [CGI-C]). The Wilcoxon signed-rank test was applied to the modified CGI-C value to test the statistical significance of the CGI-C at subsequent visits (visit 2, visit 3, and visit 4). Testing was done both with and without using the LOCF method. All analyses were defined as significant when the two-sided *p*-value was < 0.05. Data collection, trimming, and statistical analyses were performed using SAS version 9.4 (SAS Institute Inc., Cary, North Carolina, USA).

## Results

### Study populations

**Fig. 1:**
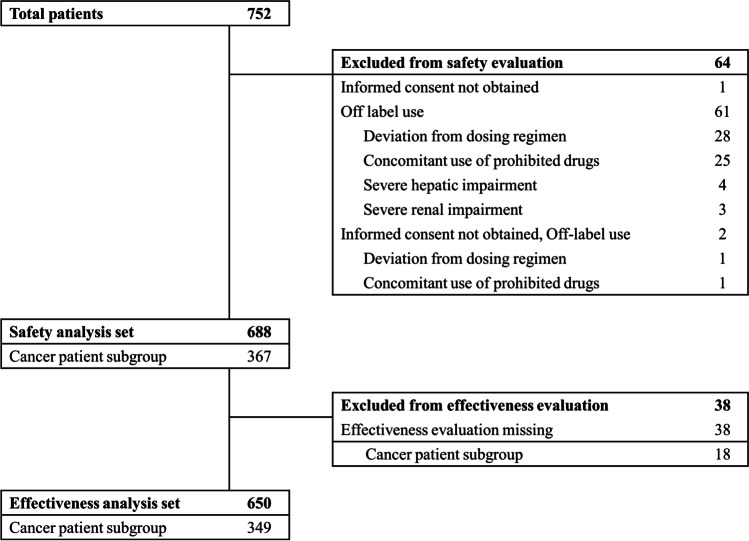
Flow diagram of the patients who were enrolled in the study

This study included 752 patients who had uncontrolled chronic pain including cancer pain with or without the use of other analgesics. From this group, 64 patients were excluded from safety evaluation, leaving 688 patients, of whom 367 were cancer patients (Fig. 1). The initial course of this study recruited patients with other chronic pain; however, in this study, we analyzed only the 367 patients with cancer pain. Of these, 18 were excluded from the efficacy evaluation owing to the lack of efficacy data; finally, 349 cancer patients completed the assessment. Table 1 lists the baseline demographics and clinical characteristics of these patients (*n* = 367). The median age was 65 years (range, 20–90 years), and approximately 70% were aged > 60 (68.7%). At the baseline evaluation (visit 1), the mean NRS score ± SD was 7 ± 0.84. There were 53 patients (15.2%) with neuropathic pain, 270 (77.6%) had no neuropathic pain, and 25 (7.2%) were not investigated. There were 265 (72.2%) opioid-naïve patients with no history of analgesics, and 102 patients (27.8%) who were previously taking the analgesics discontinued them immediately before the treatment. Of these 102 patients, 96 (98%) used opioid analgesics, among whom 71 (74.0%) were reported to have switched the treatment because of the ineffectiveness of the prior analgesics (Table 2). The most common primary cancer type was lung cancer (14.7%), followed by gastric cancer (12.8%), hepatobiliary cancer (12.3%), pancreatic cancer (10.6%), colorectal cancer (10.4%), and breast cancer (9%). Hematologic malignancies such as multiple myeloma, lymphoma, and leukemia were also included (8.7%).

**Table 1:** Baseline demographic and clinical characteristics of the cancer patients

Characteristic		No. of patients (*n* = 367)	
		*N*	(%)
Age, years	Mean ± SD	64.63 ± 11.39	
	Median	65	
Sex	Male	225	(61.3)
	Female	142	(38.7)
Pregnancy	Pregnant	0	(0)
	Not pregnant	142	(38.7)
	N/A	225	(61.3)
Breastfeeding	Yes	0	(0)
	No	142	(38.7)
	N/A	225	(61.3)
Type of care	Outpatient	294	(80.1)
	Inpatient	73	(19.9)
NRS at baseline	Number	349	
	Mean ± SD	7 ± 0.84	
	Median	7	
Neuropathic pain at baseline	Yes	53	(15.2)
	None	270	(77.6)
	Unknown	25	(7.2)
Hepatic impairment	Absent	329	(89.7)
	Present	38	(10.4)
Renal impairment	Absent	355	(96.7)
	Present	12	(3.3)
Concomitant medication	Absent	14	(3.8)
	Present	353	(96.2)
Analgesic discontinued immediately before study treatment	Absent	265	(72.2)
	Present	102	(27.8)
Malignant neoplasms		367	(100.0)
Lung cancer		54	(14.7)
Gastric cancer		47	(12.8)
Hepatobiliary cancer		45	(12.3)
Pancreatic cancer		39	(10.6)
Colorectal cancer		38	(10.4)
Breast cancer		33	(9.0)
Hematologic malignancy		32	(8.7)
Head and neck cancer		12	(3.3)
Soft tissue sarcoma		9	(2.5)
Esophageal cancer		6	(1.6)
Others		67	(18.2)

*SD*, standard deviation; *NRS*, Numeric Rating Scale; *N/A*, not applicable

**Table 2:** Prior opioid analgesic treatment and discontinuation reason

	No. of patients used opioid analgesics (*n* = 96)	
	*N*	(%)
Prior opioid analgesics^1)^		
Morphine	1	(1.0)
Oxycodone	16	(16.7)
Naloxone + oxycodone	33	(34.4)
Fentanyl	13	(13.5)
Buprenorphine	1	(1.0)
Hydromorphone	4	(4.2)
Codein	4	(4.2)
Codein + ibuprofen + paracetamol	17	(17.7)
Pethidine	1	(1.0)
Tramadol	3	(3.1)
Tramadol + paracetamol	14	(14.6)
Reason for discontinuation of prior analgesics^1)^		
Ineffectiveness of the prior analgesics	71	(74.0)
Investigator’s judgment	17	(17.7)
Patient requirement	6	(6.3)
Others^2)^	3	(3.1)

1) Duplicate response, 2) other specify; for convenience of prescription (2), adverse event (1)

### Tapentadol ER treatment

During this study, the mean duration of exposure to tapentadol ER was 73.8 days, and the daily dose was 126.7 ± 53.88 mg/day (mean ± SD). The majority of patients (71.4%) started tapentadol ER with a dose of 50 mg twice daily, 20.2% of patients were treated with a dose of 100 mg twice daily, and 3.8% were treated with 150 mg twice daily. At visits 2 and 3, 349 and 218 patients were assessed, respectively, and 90 patients completed the extended visit 4.

### Efficacy

**Fig. 2:**
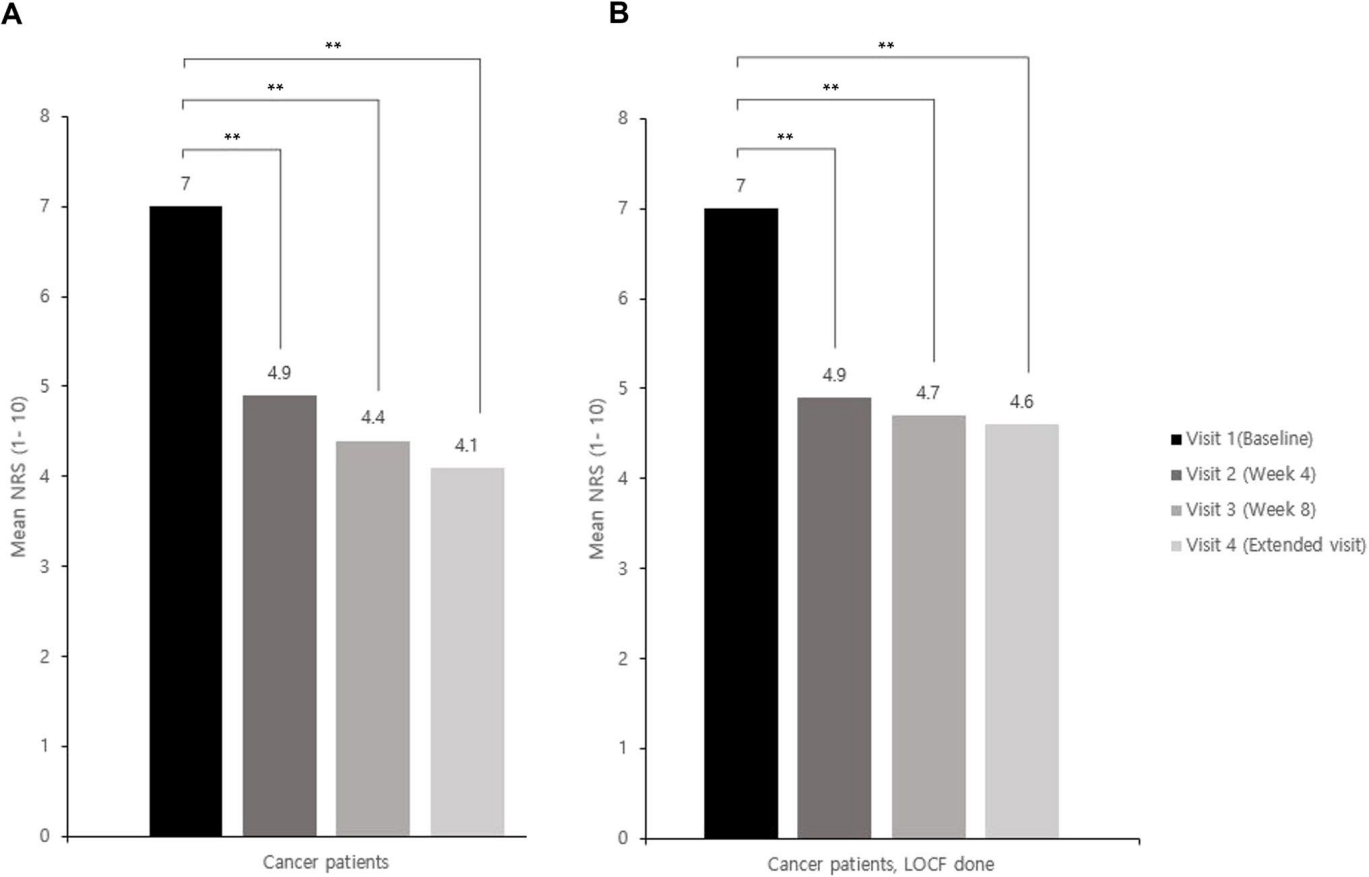
Changes in mean pain intensity in the NRS score at each visit compared to baseline for cancer patients. Data are represented as the mean of pain intensity in the NRS, which was collected at baseline and during each visit. Patients were enrolled at baseline (*n* = 349). **A** There were 328, 204, and 87 patients at visit 2, visit 3, visit 4, respectively, and the NRS score was evaluated at each visit. *p*-values are based on the Wilcoxon signed-rank test. **B** For the LOCF group, the LOCF method was performed with each patient’s NRS value at visit 2 or later visits. NRS, Numeric Rating Scale; LOCF, last observation carried forward; ** *p* < 0.0001

**Fig. 3:**
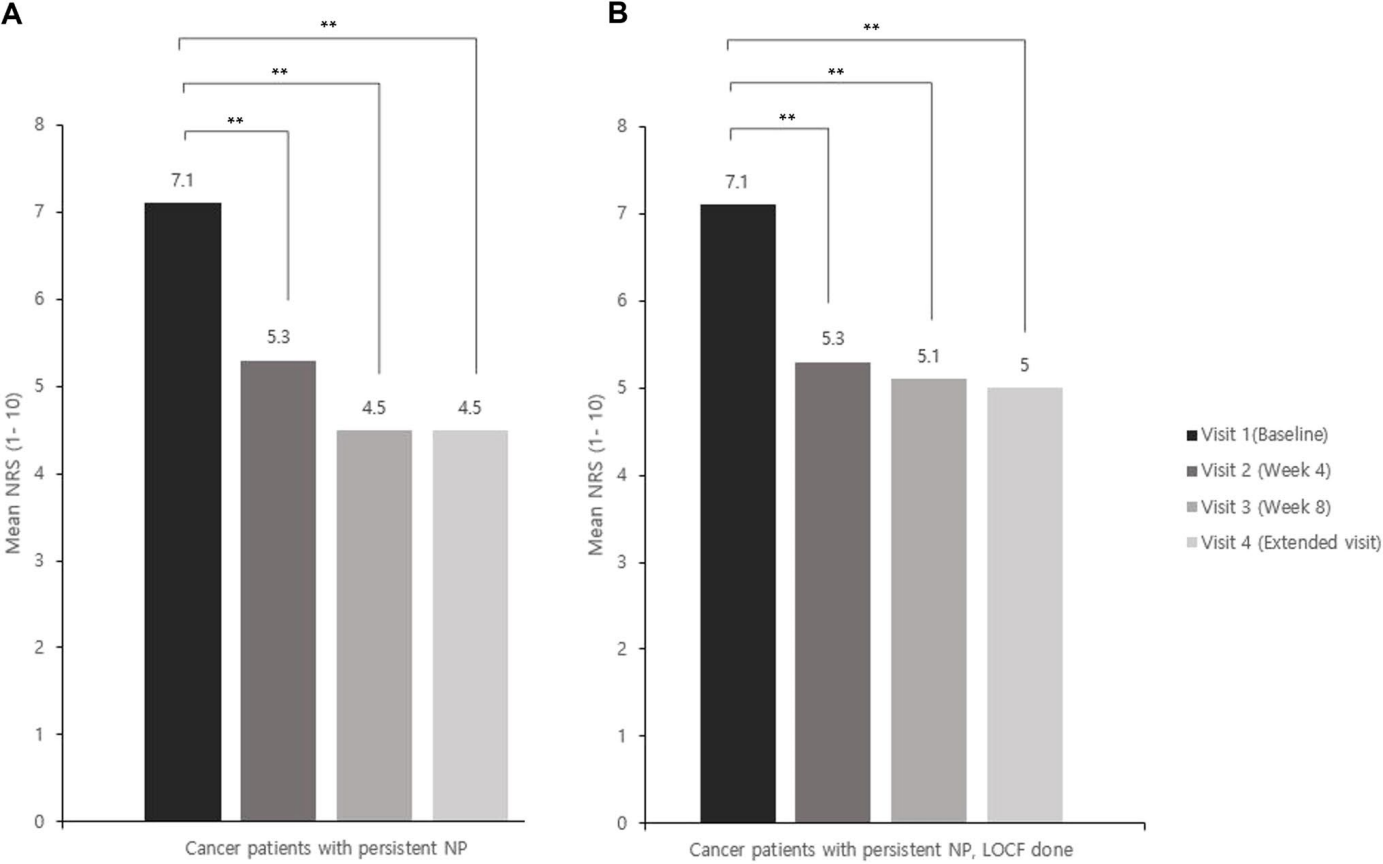
Changes in mean pain intensity in the NRS score at each visit compared to baseline for cancer patients who had neuropathic pain at visit 1 and no change in subsequent visits. Patients were enrolled at baseline (*n* = 48). **A** There were 45, 31, and 14 patients at visit 2, visit 3, visit 4, respectively, and the NRS score was evaluated at each visit. *p*-values are based on the Wilcoxon signed-rank test. **B** For the LOCF group, the LOCF method was performed with each patient’s NRS value at visit 2 or later visit. NRS, Numeric Rating Scale; LOCF, last observation carried forward; ** *p* < 0.0001

From the baseline evaluation, the NRS results for the four visits were 7 ± 0.84, 4.9 ± 2.06, 4.4 ± 2.18, and 4.1 ± 2.02, respectively. The mean pain intensity at each visit was significantly decreased compared to the baseline (Fig. 2A). In addition, it was observed that even if the missing values were corrected using the LOCF method at each visit, there was a significant reduction (Fig. 2B). Among the 326 patients at visit 2, the PID in 132 (40.5%) improved by > 30%, and in 73 (22.4%), it improved by > 50%. Of the 204 patients at visit 3, the PID in 110 (53.9%) improved by > 30%, and in 64 (31.4%), it improved by > 50%. At baseline evaluation (visit 1), there were 53 patients with neuropathic pain, among whom 48 patients answered that they still had neuropathic pain at visit 2. In fact, only 5 patients (9.4%) showed improvement in neuropathic pain; however, the mean pain intensity was significantly decreased in those 48 patients (Fig. 3).

**Fig. 4:**
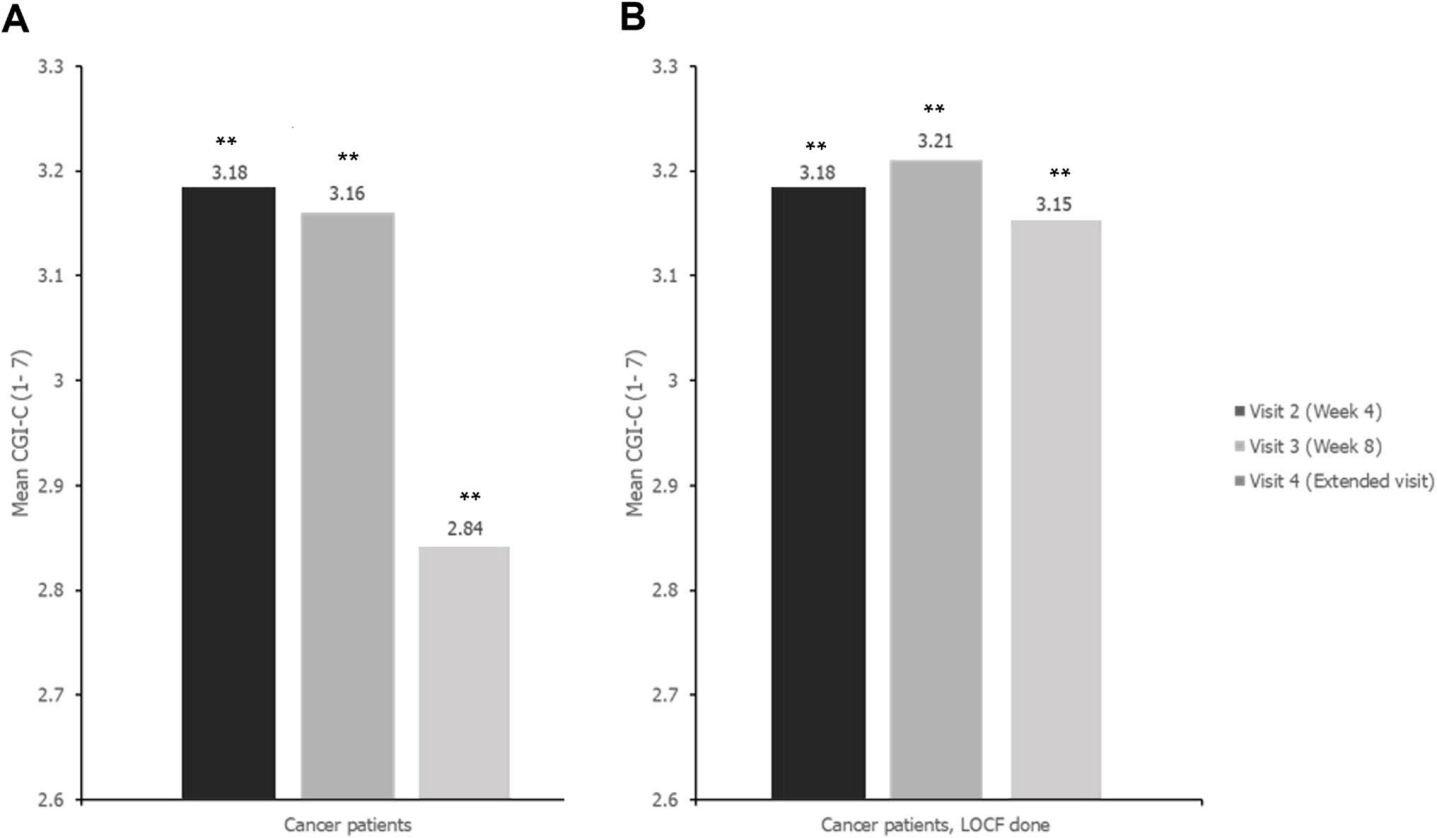
The CGI-C of cancer patients (*n* = 349) at each visit compared to baseline. Data are represented as the mean of the CGI-C, which was collected at each visit. The CGI-C ranges from 1 = “very much improved” to 7 = “very much worse.” Patients were enrolled at baseline (*n* = 349). **A** There were 349, 218, and 90 patients at visit 2, visit 3, and visit 4, respectively, and the CGI-C was evaluated at each visit. *p*-values are based on the Wilcoxon signed-rank test. The Wilcoxon signed-rank test was performed on the recalculated CGI-C values that ranged from − 3 = “very much worse” to 3 = “very much improved,” to make “no change” to be 0. **B** For the LOCF group, the LOCF method was performed with each patient’s CGI-C value at visit 2 or later visits. CGI-C, clinical global impression change; LOCF, last observation carried forward; ** *p* < 0.0001

Of the patients examined at each visit after baseline, 91.9%, 88.5%, and 97.8% of the patients, respectively, showed improvement in pain management (no change or greater, 0 to 4) based on the CGI-C assessment. The effectiveness in each case also showed statistically significant results (*p* < 0.0001, *p* < 0.0001, and *p* < 0.0001, respectively; Fig. 4). These results indicated that tapentadol ER improved the QoL of cancer patients, and the CGI-C was reflective of the patients’ QoL.

### Safety

During the study period, 96 patients (26.2%) had an adverse drug reaction, of which the most common was constipation (*n* = 14; 3.8%), followed by decreased appetite (*n* = 9; 2.5%) and esophageal pain (*n* = 7; 1.9%) (Table 3). Over the entire course of the study, 18 patients (4.9%) discontinued tapentadol ER owing to adverse drug reactions. Of those, three patients (0.8%) had serious adverse events, which were abdominal distension, asthenia, and pneumonia, respectively. In general, the majority of patients tolerated treatment with tapentadol ER.

**Table 3:** List of adverse drug reactions

Adverse drug reactions	No. of patients (*n* = 367)	
	*N*	(%)
Constipation	14	(3.8)
Decreased appetite	9	(2.5)
Esophageal pain	7	(1.9)
Dyspepsia	6	(1.6)
Nausea	6	(1.6)
Dizziness	4	(1.1)
Pyrexia	4	(1.1)
Productive cough	4	(1.1)
Neutropenia	3	(0.8)
Cough	3	(0.8)
Diarrhea	3	(0.8)
Upper respiratory tract infection	3	(0.8)
Insomnia	3	(0.8)
Pruritus	3	(0.8)
Peripheral sensory neuropathy	3	(0.8)
Neuropathy peripheral	2	(0.5)
Vomiting	2	(0.5)
Sleep disorder	2	(0.5)
Anxiety	2	(0.5)
Cystitis noninfective	2	(0.5)
Pneumonia	2	(0.5)
Others*	23	(6.3)

^*^Urethritis, stoma site infection, herpes zoster, hyperkalaemia, biliary tract infection, colostomy, glucose tolerance impaired, pulmonary oedema, erythema, pneumothorax, pneumonitis, asthenia, chest discomfort, dyspnoea, pleural effusion, peripheral circulatory failure, oropharyngeal pain, oral candidiasis, cancer pain, peripheral ischaemia, oedema,bronchitis, neutrophil count decreased, alanine aminotransferase increased, dysuria, cholecystitis infective, deep vein thrombosis, febrile neutropenia, bronchial obstruction, lung neoplasm malignant, headache, ileus, hypomagnesaemia, urinary tract infection, abdominal distension, agitation, anemia, blood alkaline phosphatase increased, hypocalcaemia, hypertension

## Discussion

This study showed that tapentadol ER significantly alleviated pain in opioid-naïve or opioid-resistant patients, and it also significantly improved their QoL. Of these patients, 40.5% showed a pain relief effect of > 30% and 22.4% showed a pain relief effect of > 50% at visit 2. Furthermore, 53.9% of the patients showed a pain relief effect of > 30%, and 31.4% of the patients showed a pain relief effect of > 50% at visit 3 (Fig. 2). As mentioned above, evaluation at visits 2, 3, and 4 after baseline showed that 91.9%, 88.5%, and 97.8% of patients, respectively, had improved QoL as evaluated by the CGI-C, indicating significant improvement (Fig. 4). However, we do not know clearly whether or not neuropathic pain was reduced, because the investigation on neuropathic pain was not quantified in this study. Among the 53 patients who answered that they had neuropathic pain at baseline, 48 answered that they had neuropathic pain at visit 2; nevertheless, the pain relief observed in these 48 patients was statistically significant (Fig. 3). These results suggest that neuropathic pain improved even in patients who answered that they still had neuropathic pain. To clarify this, a survey should to be conducted to quantify the neuropathic pain.

There are other limitations to this study. As in many studies of pain in cancer patients, many patients were lost to follow-up, and others discontinued treatment for various other reasons related to cancer. To overcome this limitation and obtain more accurate results, this study used the LOCF method to correct for missing values in statistical analysis. In addition, more accurate evaluation and corresponding results could be obtained if the patients’ QoL was evaluated using subjective indicators, such as the Hospital Anxiety and Depression Scale, Marburger questionnaire on habitual health findings (Marburger Fragebogen zum habituellen Wohlbefinden), and 12-Item Short Form Survey as well as the CGI-C [33–36].

Tapentadol ER was well-tolerated in opioid-naïve or opioid-resistant patients. Only 4.9% of patients discontinued treatment owing to adverse drug reactions during the entire study, and only 0.8% of them had serious adverse drug reactions. The safety profile of tapentadol ER indicated that it could be used flexibly for the treatment of cancer pain, and significant improvements can be obtained with few side effects.

Previous reports have shown that many cancer patients receive treatment to manage their pain; however, that treatment is often inadequate, meaning the patients endure anti-cancer treatment without adequate pain control. The first reason for this inadequacy is that oncologists may lack awareness regarding the patient’s pain owing to their focus solely on the treatment of cancer, and the second reason is that cancer pain is a mixed type of pain, composed of nociceptive and neuropathic pain. Even though there are many previous studies, there are no reports on any specific opioid being superiorly effective [7, 37, 38]. Therefore, pain must be controlled by separate mechanisms, which inevitably increase the usage of multiple drugs, further leading to decreased patient adherence to the regimen [39]. However, tapentadol ER regulates pain by two mechanisms: as a mu-opioid receptor agonist and as noradrenaline reuptake inhibitor [40]. Hence, its pain control effect is superior to that of other strong opioids, such as morphine, as demonstrated in several clinical trials. In addition, the adverse effects of tapentadol ER have been reported to be fewer than those of other strong opioids [23, 24]. Therefore, tapentadol ER will continue to be one of the most preferred drugs in the future. However, to date, data from only small-scale clinical trials are available; therefore, large-scale studies are required. This study is meaningful in that it is an extensive, real-world, large-scale study involving 349 patients with cancer who were treated with tapentadol ER regardless of prior opioid use. Our results showed that the pain of the patients treated with tapentadol was significantly reduced, the drug-related adverse reactions were very few, and the drug was well tolerated during the entire 6-month period of the study.

In conclusion, this study demonstrated that tapentadol ER can be readily substituted for other strong opioids for pain control in cancer patients. It has good compliance and can be a promising analgesic in the future for cancer patients with neuropathic pain.

## Data Availability

Due to the nature of this research, participants of this study did not agree for their data to be shared publicly, so supporting data is not available.
